# The neurophysiological basis of bruxism

**DOI:** 10.1016/j.heliyon.2021.e07477

**Published:** 2021-07-03

**Authors:** Andrisani Giovanni, Andrisani Giorgia

**Affiliations:** a Matera, via della Croce 47, Italy; b Ezelsveldlaan 2, 2611 rv, Delft, the Netherlands

**Keywords:** Bruxism, Mesencephalic trigeminal nucleus, Rhythmic masticatory muscle activity, Ventrolateral preoptic nucleus

## Abstract

Mesencephalic trigeminal nucleus (MTN) neurons innervate the stretch receptors of the jaw elevator muscles and periodontal ligament mechanoreceptors, Bruxism activates the MTN. We analyzed how MTN cells are structured, their anatomy and physiology, and the effects of their activation.

To induce and maintain sleep, gamma-aminobutyric acid (GABA), an inhibitor neurotransmitter, is released from the ventro-lateral preoptic area of the hypothalamus and acts on the ascending reticular activating system (ARAS) nuclei. The GABA neurotrasmitter induces the entry of chlorine into cells, hyperpolarizing and inhibiting these. MTN cells, on the contrary, are depolarized by GABA, as their receptors are activated upon GABA binding. They “let out” chlorine and activate ARAS cells. MTN cells release glutamate, an excitatory neurotransmitter onto their target cells, in this case onto ARAS cells. During wakefulness, ARAS activation causes cerebral cortex activation; instead, during sleep (sleep bruxism), ARAS activation avoids an excessive reduction in ARAS neurotransmitters, including noradrenaline, dopamine, serotonin, acetylcholine and glutamate. These neurotransmitters, in addition to activating the cerebral cortex, modulate vital functions such as cardiac and respiratory functions. Polysomnography shows that sleep bruxism is always accompanied by cardiac and respiratory activation and, most importantly, by brain function activation. Bruxism is not a parafunction, and it functions to activate ARAS nuclei.

## 1 Introduction

In 2013, consensus was obtained on a definition of bruxism as repetitive masticatory muscle activity characterized by clenching or grinding of the teeth and/or by bracing or thrusting of the mandible, and specified as either sleep bruxism or awake bruxism [1]. Bruxism directly involves the teeth and masticatory muscles; therefore, the trigeminal nerve, whose central nuclei include the mesencephalic trigeminal nucleus (MTN), the main sensory nucleus and the trigeminal spinal nucleus [2].

## 2 Methods

We performed a thorough search of the main search engines (PubMed; PubMed Central; Medline Plus; The Cochrane Library; Medscape; NLM Gateway; Google Scholar) regarding everything that is known about the mesencephalic trigeminal nerve.

## 3 Results

The mesencephalic trigeminal nerve (MTN) is not a nucleus but a ganglion and is the only intraneuraxial ganglion. The MTN is mostly composed of large, glutamatergic, pseudounipolar cells [3], characterized by the presence of GABA-A receptors. These receptors depolarize upon the binding of gamma-aminobutyric acid (GABA), and they allow only chlorine ions to exit the cells [4, 5, 6], similar to prenatal GABAergic neurons [7, 8, 9, 10]. Normally, GABA increases chlorine ion conductance, which, upon entering the cell, makes the postsynaptic membrane potential more negative and thereby decrease the probability that an action potential will be induced.

The peripheral branches of MTN neurons innervate the stretch receptors of the jaw elevator muscles and periodontal ligament mechanoreceptors [11]. The central branches provide glutamatergic signals to the trigeminal motor nucleus (Mo5) [3]. They project upwards toward the orexinergic hypothalamic nuclei [12] and laterally toward the adjacent locus coeruleus (LC) nucleus (the main source of noradrenergic fibers), the reticular parvocellular area, the mesencephalic reticular formation, the dorsal raphe nucleus (DRN, the main source of serotoninergic fibers), and to the latero-dorsal tegmental (LDT) nucleus [11, 13] (the main source of cholinergic fibers in the brainstem). The central branches of MTN neurons then descend into the latero-tegmental area, forming the Probst tract, finally terminating at the caudal trigeminal nucleus and up to the first segments of the spinal cord [14, 15].

It should be noted that the efferent branch of these cells innervates only two structures: the chewing muscles and the periodontium. Therefore, MTN is activated with the opening and closing of the mouth and with dental contact, implying that the MTN is particularly active during bruxism. In addition, MTN nerve endings, afferent to the central nervous system, release only glutamate and therefore are excitatory. These MTN cells release glutamate to the ascending reticular activating system (ARAS) nuclei.

ARAS nuclear cells release neurotransmitters (NTs), including noradrenaline, dopamine, serotonin, acetylcholine, and glutamate, with a small proportion released directly into the cerebral cortex and exciting it, while the majority is released into the thalamus and basal forebrain. This, in turn, excites the cerebral cortex [16, 17, 18, 19, 20, 21]. Some afferent MTN fibers innervate the trigeminal motor nucleus (Mo5).

MTN pseudounipolar cells are equipped with prenatal GABA-A receptors that activate ARAS cells [15, 16, 17, 18, 19, 20] and are activated by GABA [4, 5, 6], which is released to induce and maintain sleep.

To induce and maintain sleep, the central nervous system inhibits ARAS nuclei through the hypothalamic ventro-lateral preoptic (VLPO) nucleus and other nuclei, such as the thalamic reticular nucleus and nucleus accumbens [22]. The neurons of these nuclei use inhibitor neurotransmitters such as GABA and galanin, and their connections are directed toward the ARAS nuclei neurons, particularly the orexinergic neurons of the lateral hypothalamus, the histaminergic neurons of the tubero-mammillary nucleus (TMN), the serotonergic neurons of the DRN, the noradrenergic neurons of the LC, and the cholinergic neurons of the basal forebrain (BFC), LDT, and pedunculo-pontine tegmentum (PPT). When hypothalamic GABA is released on ARAS nuclei neurons, their neurotransmitters, including serotonin, orexin, and noradrenaline, among others, are neither produced nor released [23, 24].

## 4 Interpretation

GABA, released by the hypothalamic nucleus VLPO, activates the MTN which, in turn, can activate the ARAS nuclei. The activation of ARAS nuclei by MTN during sleep prevents an excessive reduction in the aforementioned ARAS neurotransmitters that, in addition to activating the cerebral cortex, also support cardiac and respiratory function during wakefulness and sleep. Therefore, MTN and sleep bruxism have an important protective role.

We can verify ARAS nuclear activation and its association with sleep bruxism experimentally. During polysomnography, sleep bruxism is identified through several means: by electromyography through rhythmic masticatory muscle activity (RMMA) that represents the polysomnographic manifestation of sleep bruxism [1]; with an electrocardiogram as the heart rate increases in association with sleep bruxism [25, 26]; through an increase in breathing rate [27]; and, most importantly, in the electroencephalogram, which shows an increase in cerebral cortex activity during sleep bruxism [28, 29, 30, 31].

## 5 Therapies

Sleep bruxism cannot be eliminated because this would also eliminate GABA, and without GABA there is no sleep. Very little is known about the complex relationship between drugs and GABA release in the context of sleep; however, we cannot help but notice that some GABA agonist drugs (alcohol, phenethylamines (amphetamines and methylphenidate), heroin, anticonvulsants, and selective serotonin reuptake inhibitors) favor sleep bruxism while some GABA antagonists (clonidine, levodopa, clonazepam, gabapentin, hydroxyzine, and dopamine agonists) reduce it [32, 33].

## 6 Conclusion

Bruxism stimulates ARAS nuclei, so it is not a parafunction. Its function is to activate the production of ARAS neurotransmitters that stimulate the cerebral cortex, as confirmed by chewing studies [34, 35, 36, 37]. When bruxism no longer works properly (e.g., in elderly edentulous patients without dentures), individuals are at higher risk of developing dementia [38, 39, 40, 41, 42, 43, 44, 45].

## Declarations

### Author contribution statement

All authors listed have significantly contributed to the development and the writing of this article.

### Funding statement

This research did not receive any specific grant from funding agencies in the public, commercial, or not-for-profit sectors.

### Data availability statement

Data included in article/supplementary material/referenced in article.

### Declaration of interests statement

The authors declare no conflict of interest.

### Additional information

No additional information is available for this paper.
